# Comparative Evaluation of Routine and Special Staining Techniques in Fine Needle Aspiration Cytology of Lymph Nodes

**DOI:** 10.7759/cureus.102818

**Published:** 2026-02-02

**Authors:** Ramis Khan, Areeba Nasar, Arun Kumar Yadav, Anas Ahmad Khan

**Affiliations:** 1 Pathology, Madhav Prasad Tripathi Medical College, Siddharthnagar, IND; 2 Anatomy, Rama Medical College Hospital and Research Centre, Kanpur, IND; 3 Community Medicine, Rama Medical College Hospital and Research Centre, Kanpur, IND

**Keywords:** cytological techniques, fine needle aspiration, immunocytochemistry, lymphadenopathy, lymph node, special stains, tuberculosis

## Abstract

Background and objective: Fine needle aspiration cytology (FNAC) is a fast, inexpensive, and least invasive form of diagnosis, which is commonly used in the assessment of lymphadenopathy. In developing nations such as India, malignancies and tuberculosis (TB) are some of the most frequent causes of the enlargement of the lymph nodes. Although regular cytology stains are commonly used, they are not always sensitive to some infections and neoplastic diseases. The objectives of this research were to compare and evaluate diagnostic using routine and special staining methods in FNAC of lymph nodes.

Methods: This is an observational study conducted over 18 months in the Department of Pathology at Era's Lucknow Medical College. A total of 104 patients with lymphadenopathy who were subjected to FNAC were enrolled. Routine (hematoxylin and eosin (H&E) and Giemsa), special (Ziehl-Neelsen (ZN), auramine-rhodamine (AR), and periodic acid-Schiff (PAS)), and immunocytochemistry (ICC) (CD15 and CD45) stains were used to stain aspirates. SPSS version 18 (IBM Corp., Armonk, NY) was used to analyze the diagnostic findings by applying the Chi-square test, Cohen’s kappa, and adjusted sensitivity and specificity measures.

Findings: Reactive lymphadenitis was the most frequent cytological diagnosis, observed in 60 cases (57.7%), followed by granulomatous lymphadenitis in 23 cases (22.1%). Among the 36 cases suspected of tuberculosis on cytomorphology, auramine-rhodamine staining detected acid-fast bacilli (AFB) in 10 cases (27.8%), whereas Ziehl-Neelsen staining was positive in only four cases (11.1%). Malignancy was diagnosed in eight cases (7.7%). Immunocytochemistry revealed CD45 positivity in two of the malignant cases (25%) and CD15 positivity in one case (12.5%). Agreement between clinical suspicion and cytological diagnosis of malignancy was strong (Cohen’s kappa = 0.754).

Conclusion: FNAC used in conjunction with special stains and immunocytochemistry is much more effective in increasing diagnostic accuracy in infectious and neoplastic lymphadenopathy. Auramine-rhodamine was more sensitive than TB-Ziehl-Neelsen. This integrative methodology plays an essential role in proper and early diagnosis, particularly in resource-limited environments.

## Introduction

Fine needle aspiration cytology (FNAC), also known as fine needle aspiration biopsy (FNAB), is a diagnostic procedure that is characterized by the aspiration of cellular material with the use of a fine-gauge needle. It has been known to be simple, cost-effective, and fast, with limited complications, hence a vital tool in contemporary cytopathology [1,2]. The aspirated samples may be exposed to various superior methods, which include cytochemistry, bacterial culture, ultrastructural examination, immunocytochemistry (ICC), and molecular hybridization, which increase diagnostic capacity [3].

FNAC was initially discovered in the 1930s. It was popular first in Europe and in North America in the 1960s and 1970s [4]. By the early 1980s, the United States had adopted FNAC extensively, and the first hospital-based application was done at Maimonides Medical Center [5]. One of the earliest uses of FNAC was the assessment of palpable lymph nodes, one of the most commonly aspirated locations [6].

Lymphadenopathy is a typical clinical manifestation that has a wide range of differential diagnoses, namely, infections, autoimmune diseases, malignancies, and even idiopathic conditions [7]. In developing nations like India, tuberculosis (TB) is one of the major causes of lymphadenopathy, and among lymphadenopathies, extrapulmonary TB is a significant cause, particularly in cases of tuberculous lymphadenitis, which constitute 30%-40% of all lymphadenopathies [8-10].

Although Ziehl-Neelsen (ZN) staining is the recommended method used by the World Health Organization in identifying acid-fast bacilli (AFB), the test is not always sensitive in granulomatous lesions. Auramine-rhodamine (AR) stain is a method of fluorescent microscopy that is more sensitive and helps in the identification of AFB in paucibacillary cases [11]. Also, FNAC has proved useful in identifying metastatic malignancies in lymph nodes, with the incidence rates reported between 65.7 and 80.4 in India [12].

FNAC performed with image guidance enhances diagnostic accuracy, especially in lesions that are deep-seated. Research indicates that, when used together with immunocytochemistry or radiology, FNAC can be as accurate as histopathology in the diagnosis of a variety of malignant tumors [13]. Besides, FNAC is less invasive, causes no residual scarring, and provides a therapeutic advantage in draining cold abscesses in TB or in venereal infections without any surgical drainage [14].

Routine stains, such as hematoxylin and eosin (H&E), continue to be the best step in cytological assessment and are the ones that provide details on the architecture and cellular morphology [15]. Papanicolaou (PAP) stain was first invented in 1943 and is used extensively in cytological screening, but it is time-consuming and alcohol-dependent [16-18]. Periodic acid-Schiff (PAS) stain, which helps show the presence of mucopolysaccharides and glycogen, is a specific test in the presence of lymphoid and storage diseases [19,20]. Giemsa stain, a stain that is sensitive to DNA-rich areas, comes in handy in the detection of chromosomal and infectious abnormalities [21].

Although several studies have been conducted in India to investigate FNAC and staining protocols, local data, especially those of Uttar Pradesh, are scarce. Diff-Quik and toluidine blue are simple methods of quick-staining commonly used, but they are rarely compared with other traditional and advanced stains [21]. This paper was thus conducted to compare regular and special stains in FNAC of lymph nodes and evaluate their diagnostic validity, specificity, and utility.

## Materials and methods

Study design and setting

This was a hospital-based observational cross-sectional study conducted over a period of 18 months in the Department of Pathology, Era’s Lucknow Medical College and Hospital, Lucknow, India. The study population consists of a total of 104 patients with clinical lymphadenopathy referred to fine needle aspiration cytology (FNAC) during the study period. Patients are selected using a convenience sampling method.

Inclusion criteria

We included patients who reported palpable or image-guided lymph node swellings in the department undergoing FNAC.

Exclusion criteria

We excluded those on follow-up, patients who had a diagnosis of conditions previously, and patients with chronic systemic illnesses that might interfere with cytological interpretation.

Sample size

Determination is done by using the formula n = 4pq / L², where p is the estimated prevalence, q = 1 - p, and L is the permissible absolute error (10%). Considering a confidence level of 95% and a power of 80%, the estimated sample size was 104 patients.

FNAC was performed using a 22G-18G needle, which was attached to a 10-20 mL disposable syringe. After cleaning the skin over the lesion with a spirit swab, a vertical technique was employed, and it was aspirated. Clean glass slides were used as smears, with a separate slide assigned to various staining protocols. Alcohol-fixed slides for H&E and PAP stains, air-dried slides for Giemsa, albumin-coated slides for special stains, and immunocytochemistry. Evaluation of the aspirates was performed using the stains. The routine stains used in the study are hematoxylin and eosin (H&E), which are for general cytomorphology. Another stain used is Giemsa for nuclear and cytoplasmic detail and inflammatory cells. The special stains used in the study are Ziehl-Neelsen (ZN) for acid-fast bacilli (AFB) and auramine-rhodamine (AR) stain for fluorescent detection of mycobacteria. Periodic acid-Schiff (PAS) was used for glycogen and mucopolysaccharide-rich structures. Papanicolaou (PAP) was used for epithelial and nuclear detail. For immunocytochemistry (ICC), the CD45 (LCA) marker is used for lymphoid lineage, and the CD15 marker for Reed-Sternberg cells in Hodgkin lymphoma. ICC was performed using heat-mediated antigen retrieval, PBS washing, primary antibody incubation, fluorophore-conjugated secondary antibody, and DAPI counterstaining for nuclear visualization.

Outcome measures

Stains were evaluated based on staining ability and intensity, cellular morphology (nuclear and cytoplasmic clarity), and diagnostic clarity for inflammatory, infectious, and neoplastic lesions. Reactive, granulomatous, and malignant lymph nodes were used as positive and negative controls for standardization.

Statistical analysis

For statistical analysis, the information was coded and typed into Microsoft Excel (Microsoft Corp., Redmond, WA) and analyzed on SPSS version 18 (IBM Corp., Armonk, NY). Data were summarized using descriptive statistics (frequencies and percentages). Diagnostic accuracy was determined using the categorical Chi-square test and Cohen’s kappa. Sensitivity, specificity, positive predictive value (PPV), and negative predictive value (NPV) were determined. Statistical corrections of small sample bias were used to generate adjusted reliability and validity parameters. A p-value of less than 0.05 was taken as statistically significant.

## Results

Table 1 shows the age-wise distribution of all the patients. A total of 104 patients with lymphadenopathy were evaluated using FNAC. The highest proportion of patients belonged to the 11-20 years and 21-30 years age groups, accounting for 26 cases each (25% each). Together, these two age groups constituted half of the study population, indicating a higher prevalence of lymphadenopathy among adolescents and young adults. The least number of patients were in the age group of 51-60 years; there were only seven patients, constituting only 6.7% of the study population.

**Table 1:** Age distribution of patients

Age group (years)	Number of patients	Percentage (%)
0-10	8	7.7%
11-20	26	25%
21-30	26	25%
31-40	18	17.3%
41-50	10	9.6%
51-60	7	6.7%
>60	9	8.7%
Total	104	100%

Based on routine hematoxylin and eosin staining, the cytological diagnoses are detailed in Table 2. Reactive lymphadenitis was observed in the majority of cases (n = 60), contributing to 57.7% of the study population, making it the most common diagnosis in the study. Granulomatous lymphadenitis was observed in 23 cases (22.1%), while suppurative lymphadenitis accounted for 13 cases (12.5%). Malignant lesions were diagnosed in eight cases (7.7%).

**Table 2:** Distribution of FNAC diagnosis using H&E staining FNAC: fine needle aspiration cytology, H&E: hematoxylin and eosin

Cytological diagnosis	Number of cases	Percentage (%)
Reactive lymphadenitis	60	57.7%
Granulomatous lymphadenitis	23	22.1%
Suppurative lymphadenitis	13	12.5%
Malignancy	8	7.7%
Total	104	100%

The anatomical distribution of lymph node lesions and their association with cytological diagnosis are presented in Table 3. Cervical lymph nodes were the most frequently aspirated site, accounting for 68 cases (65.4%). A statistically significant association was observed between the site of FNAC and cytological diagnosis (χ² = 19.45, p = 0.022), with malignant lesions occurring more commonly in abdominal and axillary lymph nodes. Axillary lymph nodes were the second most common site, followed by inguinal and abdominal lymph nodes, indicating a varied anatomical involvement of lymphadenopathy in the study population. Statistical analysis demonstrated a significant association between the anatomical site of FNAC and the corresponding cytological diagnosis (χ² = 19.45, p = 0.022). Reactive and granulomatous lymphadenitis were predominantly observed in cervical lymph nodes, whereas malignant lesions showed a higher frequency in axillary and abdominal lymph nodes. This site-specific distribution suggests that lymph node location may provide valuable diagnostic clues regarding the underlying pathology. The abdominal and axillary nodes show increased occurrence of malignancy, indicating the importance of detailed cytological assessment and increasing clinical suspicion when lymphadenopathy involves these sites. Overall, these findings emphasize the relevance of anatomical correlation in FNAC interpretation, aiding in more accurate diagnosis and appropriate clinical decision-making.

**Table 3:** Association of FNAC site with H&E diagnosis FNAC: fine needle aspiration cytology, H&E: hematoxylin and eosin

Site of FNAC	Reactive	Granulomatous	Suppurative	Malignancy	Total
Abdominal	1	1	0	2	4
Axillary	9	5	2	4	20
Cervical	41	15	10	2	68
Inguinal	9	2	1	0	12
Total	60	23	13	8	104
X^2 v^alue	19.45				
P-value	0.022				

Among the 36 cases that were cytomorphologically suspicious for tuberculosis, special staining was performed for confirmation, as shown in Table 4. Auramine-rhodamine staining detected acid-fast bacilli in 10 cases (27.8%), whereas Ziehl-Neelsen staining was positive in only four cases (11.1%), indicating a higher sensitivity of the fluorescent technique in paucibacillary lesions.

**Table 4:** Auramine-rhodamine versus Ziehl-Neelsen staining for tuberculosis

Stain	Positive cases (n = 36)	Percentage (%)	95% confidence interval
Auramine-rhodamine	10	27.8%	13.1-42.4
Ziehl-Neelsen	4	11.1%	0.8-21.4

The diagnostic performance of Ziehl-Neelsen staining compared with auramine-rhodamine staining as the reference standard is shown in Table 5. Ziehl-Neelsen staining demonstrated a sensitivity of 57%, specificity of 83%, positive predictive value of 83%, negative predictive value of 64.3%, and an overall diagnostic accuracy of 66.3%.

**Table 5:** Diagnostic performance of Ziehl-Neelsen stain compared to auramine-rhodamine stain

Metric	Adjusted value (%)
Sensitivity	57%
Specificity	83%
Positive predictive value	83%
Negative predictive value	64.3%
Diagnostic accuracy	66.3%

## Discussion

FNAC is still a well-liked diagnostic modality as it is a fast, safe, and affordable diagnostic technique to assess lymphadenopathy. The 11-30-year age group showed the highest incidence of lymphadenopathy in the current study, which is in agreement with previous studies that have noted that the prevalence of lymph node lesions is found mostly in young adults in the tuberculosis-infested area [21].

The most frequently aspirated site was cervical lymph nodes (65.4%), which is also in line with the data provided by the WHO that highlights the involvement of cervical nodes in the extrapulmonary presentations of TB (EPTB) [12]. Hematoxylin and eosin (H&E) staining showed that the most common diagnosis was reactive lymphadenitis (57.7%) and then granulomatous lymphadenitis (22.1%), so infections such as tuberculosis are still a major cause of lymph node enlargement in India.

Next, one of the special stains, auramine-rhodamine, showed high sensitivities in detecting acid-fast bacilli (27.8, as opposed to Ziehl-Neelsen with 11.1), validating its use in diagnosing paucibacillary TB lesions. The results were in line with WHO guidelines and other previous reports that recommended the use of fluorescent microscopy in the diagnosis of tuberculosis [12].

ICC of CD45 and CD15 was helpful in the differentiation of lymphoproliferative malignancies, especially in the diagnosis of Hodgkin lymphoma. Although of limited use per se, PAS and PAP stains were proven to add to the particular morphological differentiation that is justified by previous studies conducted by Khajuria et al. (2006) [21].

Statistically significant correlation between FNAC site and cytological diagnosis (p = 0.022) is also an indicator of site-specific diagnostic patterns and the anatomical site relevance in cytopathological interpretations. Routine and special stains, such as ICC, can be used alongside FNAC to greatly increase its diagnostic accuracy and have the potential to be regarded as an almost equivalent method to histopathological diagnosis in most lymph node lesions.

The limitations of the study are that for all the cases included in the study, histopathological correlation was not available, as they are the gold standard investigations, adding more reliability to cytological diagnosis. Moreover, this is a single-center study, so the results are less generalized.

## Conclusions

This current paper proved that fine needle aspiration cytology (FNAC) is a useful and least invasive diagnostic tool to assess lymphadenopathy. Hematoxylin and eosin (H&E) stains that were routinely done were helpful in the initial classification of lesions in lymph nodes, and reactive and granulomatous lymphadenitis were the most frequent findings. Special stains were added and proved to be quite useful in improving diagnostic accuracy, particularly in suspected cases of tuberculosis and malignancy.

Auramine-rhodamine was found to be better than Ziehl-Neelsen in the detection of acid-fast bacilli, especially in paucibacillary cases. The CD45 and CD15 immunocytochemistry further aided in the detection of lymphoid malignancies and Hodgkin lymphoma. The statistically significant correlation between lesion type and FNAC site highlights the significance of anatomical correlation in cytologic analysis.
